# Human Umbilical Cord Mesenchymal Stem Cell-Derived Extracellular Vesicles Carrying MicroRNA-181c-5p Promote BMP2-Induced Repair of Cartilage Injury through Inhibition of SMAD7 Expression

**DOI:** 10.1155/2022/1157498

**Published:** 2022-06-24

**Authors:** Qiang Zhang, Le Cao, Shanqi Zou, Yuanling Feng, Xudong Miao, Lu Huang, Yongping Wu

**Affiliations:** ^1^Foot and Ankle Group of Orthopaedics, The Second Hospital Affiliated to Zhejiang University School of Medicine, Hangzhou 310000, China; ^2^Obstetrics Department, The Second Hospital Affiliated to Zhejiang University School of Medicine, Hangzhou 310000, China; ^3^Foot and Ankle Group of Orthopaedics, The Second Hospital of Quzhou, Quzhou 324000, China

## Abstract

The therapy role of mesenchymal stem cell- (MSC-) derived extracellular vesicles (EVs) in cartilage regeneration has been well studied. Herein, we tried to analyze the role of human umbilical cord MSC- (hUCMSC-) EVs carrying microRNA- (miR-) 181c-5p in repair of cartilage injury. After successful isolation of hUCMSCs, the multidirectional differentiation abilities were analyzed. Then, the EVs were isolated and identified. After coculture of PKH26-labled EVs with bone marrow MSCs (BMSCs), the biological behaviors of which were detected. The relationship between the predicted early posttraumatic osteoarthritis-associated miRNA, miR-181c-5p, and SMAD7 was verified. Gain- and loss-of functions were performed for investing the role of miR-181c-5p and SMAD7 in BMP-induced chondrogenesis *in vitro* and *in vivo*. hUCMSC-EVs could be internalized by BMSCs and promote the proliferative, migratory, and chondrogenic differentiation potentials of BMSCs. Additionally, miR-181c-5p could target and inhibit SMAD7 expression to promote the bone morphogenic protein 2- (BMP2-) induced proliferative, migratory, and chondrogenic differentiation potentials of BMSCs. Also, overexpression of SMAD7 inhibited the repairing effect of BMP2, and overexpression of BMP2 and miR-181c-5p further promoted the repair of cartilage injury *in vivo*. Our present study highlighted the repairing effect of hUCMSC-EVs carrying miR-181c-5p on cartilage injury.

## 1. Introduction

Cartilage, a type of connective tissue, acts as a buffer in pressure and exerts an important role in protection of joint movement; however, cartilage is hard to self-recover after injury due to the lack of nerve tissues and blood vessels [1]. Moreover, the hyaline cartilage located at the knee joint has the features of both being elastic and stiff, which adds more challenges in the repair of cartilage injury [2]. Recently, an endogenous regenerative strategy, which simultaneously promotes the recruitment of chondrocytes and bone marrow mesenchymal stem cells (BMSCs), is of great importance for cartilage regeneration [3].

Umbilical cord mesenchymal stem cells (UCMSCs) have been demonstrated to be effective in bone regeneration because of their advantages of easy collection, good differentiation capacity, and low immunogenicity [4, 5]. Interestingly, extracellular vesicles (EVs) derived from human UCMSCs have been widely used as a therapeutic approach in treating different diseases including neurological, cardiovascular, bone, cutaneous wound, and cartilage disorders [6]. What is more, hUCMSC-derived EVs (hUCMSC-EVs) carrying microRNAs (miRNAs) might be an inspiring therapeutic strategy for the clinical application of hUCMSC-EVs in disease treatment [7]. As documented earlier, miR-181c is highly expressed in hUCMSC-EVs [8]. Moreover, miR-181c is involved in the treatment of osteoarthritis characterized by damage of articular cartilage [9]. Smad7 is found to be a direct target gene of miR-181a and miR-181b in neuroinflammation [10]. However, the role of SMAD7 in repair of cartilage injury is under investigation. Downregulation of SMAD7 promotes bone morphogenetic protein 2- (BMP2-) induced chondrogenesis [11]. BMP2 is a key factor for inducing cartilage differentiation and can promote the chondrogenic differentiation of stem cells [12]. BMP2 also is a potent inducer in charge of the osteoblast differentiation in BMSCs [13]. In view of the above evidence, the present study was aimed at conducting an in-depth investigation into the repair of cartilage injury and assessing the role of hUCMSC-EVs and its underlying molecular mechanism in this process.

## 2. Materials and Methods

### 2.1. Bioinformatics Analysis

The early posttraumatic osteoarthritis-associated miRNA microarray dataset GSE99736 was obtained from the Gene Expression Omnibus database with the anterior synovial region samples 1 week after sham operation (control; *n* = 4) and 6 weeks after sham operation (recovery; *n* = 4) selected. Differentially expressed miRNAs from the two groups were analyzed utilizing the R language “limma” package with |log2FoldChange| > 0.6 and *p* < 0.05 as the criteria, followed by construction of heat maps with the help of the R language “pH&Eatmap” package.

The target genes of miRNAs were predicted by the StarBase, TargetScan, PicTar, and microT. Correlation between the candidate genes and diseases was analyzed by means of the PH&Enolyzer tool. Coexpression relationships among genes were analyzed through CAdxpedia, and the protein-protein interaction (PPI) network was analyzed utilizing the STRING database, which was visualized with the help of Cytoscape 3.5.1 software.

### 2.2. Cell Culture

HEK293 cells and human BMSCs were purchased from the American Type Culture Collection (ATCC) cell bank (Manassas, USA) and cultured in Dulbecco's modified Eagle's medium (DMEM, HyClone, Logan, UT, USA) supplemented with 10% fetal bovine serum (FBS; Gibco, Waltham, MA, USA), 100 U/mL penicillin, and 100 mg/mL streptomycin (Gibco) at 37°C, 5% CO_2_, and saturated humidity.

### 2.3. Isolation, Culture, and Identification of hUCMSCs

The study was ratified by the ethics committee of the Second Hospital Affiliated to Zhejiang University School of Medicine, and informed consent was offered by all participants prior to sample collection. For isolation and culture of hUCMSCs, human umbilical cord samples (*n* = 3) were obtained from healthy mothers after delivery and processed within 6 h after collection. Umbilical cords were first washed twice with phosphate buffer saline (PBS) replenishing penicillin and streptomycin to remove blood. The rinsed umbilical cords were cut into 3-4 cm segments in a culture dish and cultured in DMEM/F-12 (Gibco) supplemented with 10% FBS (Gibco), 1% GlutaMAX (Gibco), 100 U/mL penicillin, and 100 *μ*g/mL streptomycin (Gibco) at 37°C with 5% CO_2_ for 72 h. After that, cells were cultured in Ultra medium (Lonza, 12-725 F, Swiss) replenishing the serum analogue Ultroser™ G (259509, Life Science, New Brunswick, NJ, USA). Subsequently, half of the medium was renewed every three days, and umbilical cord tissues were removed after the presence of a fibroblast-like cell colony. Cells were detached with trypsin and passaged until 80-90% confluence. Early passaged hUCMSCs (at passages 2 to 6) were used for subsequent experimentations.

For identification of surface markers of hUCMSCs, 1 × 10^4^ cells were incubated with primary antibodies (all from BD, Franklin Lakes, NJ, USA): PE anti-human CD44 (1 : 100, 550989), PE anti-human CD151 (1 : 100, 556057), PE anti-human CD73 (1 : 100, 550257), APC anti-human CD133 (1 : 100, 566596), and PE anti-human CD34 (1 : 100, 560710) at 4°C for 30 min. After PBS washing, cells were incubated with specific secondary antibodies for 30 min (dark condition), followed by analysis of surface marker expression utilizing a FACScan flow cell flow system (Becton Dickinson, San Diego, CA, USA).

For detection of differentiation abilities of hUCMSCs, hUCMSCs were cultured in osteoblast medium (HUXUC-90021; Cyagen, San Clara, CA, USA) for 14 days or in lipogenic medium (HUXUC-90031; Cyagen) for 21 days or chondrogenic differentiation medium (HUXUC-9004; Cyagen) for 21 days. Subsequently, the differentiated cells were fixed in 4% paraformaldehyde, washed once in PBS, and then stained utilizing alkaline phosphatase (ALP) and 2% Alizarin Red (A5533, Sigma-Aldrich, St. Louis, MO, USA) to identify osteogenic differentiation of hUCMSCs. Oil Red O (O0625, Sigma-Aldrich) staining was performed to identify the adipogenic differentiation potential, while the 1% Alcian (A5268, Sigma-Aldrich) solution was to detect chondrogenic differentiation potential.

hUCMSCs treated with dimethyl sulfoxide (DMSO) were applied as the control, and the sphingomyelinase inhibitor GW4869 (10 *μ*M, Sigma) was added to the experimental group and cultured separately for 24 h. After 24 h, the conditioned medium (CM) was cultured with BMSCs. Cells were divided into control, hUCMSC-CM, and hUCMSC-GW4869-CM groups.

### 2.4. Cell Transduction

Cells were seeded into six-well plates and transduced with plasmids of the miR-181c-5p mimic, mimic negative control (NC), miR-181c-5p inhibitor, or inhibitor NC (50 nM; GenePharma, Shanghai, China) by means of Lipofectamine 2000. The packaging mixture was transduced into cells, which were collected 48 h after transduction. Overexpression or knockdown efficiency was confirmed by reverse transcription quantitative polymerase chain reaction (RT-qPCR).

### 2.5. Isolation and Identification of hUCMSC-EVs

hUCMSCs were cultured in Ultra medium (EV-free) replenishing 10% FBS (Lonza, 12-725 F) for 72 h. EVs were then collected and purified from the supernatant of CM. After 72 h, the supernatant was centrifuged at 300 g for 10 min, 2,000 g for 10 min, and 10,000 g for 30 min, respectively, at 4°C. Next, the supernatant was centrifuged at 100,000 g for 70 min at 4°C in an ultracentrifuge (Beckman Coulter, Brea, CA, USA). The supernatant was discarded, and the precipitate was resuspended with 1 mL of PBS. After centrifugation at 100,000 g for 70 min, the supernatant was discarded and the precipitate was resuspended in prechilled PBS to obtain EVs.

The obtained EVs were observed at 100 KeV under a transmission electron microscopy (TEM) (HT7830, Hitachi, Tokyo, Japan). Besides, particle size and concentration of EVs were quantified utilizing nanoparticle tracking analysis (NTA) through a NanoSight LM10 instrument (NanoSight Ltd., Minton Park, UK) at 23.75 ± 0.5°C for 60 s.

### 2.6. Uptake of hUCMSC-EVs

Isolated hUCMSC-EVs were suspended in 200 *μ*L of precooled PBS and incubated with the red fluorescent dye PKH26 (4 *μ*mol/L, MINI26-1KT, Sigma-Aldrich) at ambient temperature for 5 min. Next, the hUCMSC-EVs were incubated with BMSCs at 37°C for 24 h and fixed with 4% paraformaldehyde. The cytoskeleton was stained with a microfilament green fluorescent probe (C1033, Beyotime, Shanghai, China) for 30 min, and the nuclei were stained with 10 *μ*g/mL of Hoechst 33342 (C1025, Beyotime) for 10 min. The uptake of EVs was observed under a confocal laser scanning microscope (Zeiss LSM710, Germany), with five random fields of view taken for each sample.

### 2.7. Cy3 Fluorescent Labeling

hUCMSCs were detached with 0.25% trypsin (GIBCO) and then resuspended in Ultra medium replenishing 10% FBS to adjust the cell concentration to 1 × 10^6^ cells/well. Cy3-labeled miR-181c-5p (miR-181c-5p-Cy3) was procured from GenePharma and transfected into hUCMSCs in the light of Lipofectamine 2000 (11668 019, Invitrogen Inc., Carlsbad, CA, USA) instructions. hUCMSCs expressing Cy3-miR-181c-5p were seeded into 6-well plates and cocultured with BMSCs in Transwell chambers for 4 d. After that, the nuclei of BMSCs were stained with 10 *μ*g/mL of Hoechst 33342 (C1025, Beyotime). Finally, the fluorescence expression was observed under a confocal microscope.

### 2.8. Construction of Recombinant Adenovirus

Recombinant adenovirus was constructed with the help of AdEasy recombinant adenovirus technology. The coding region of BMP2 and SMAD7 as well as transcripts of miR-181c-5p was amplified utilizing high-fidelity PCR and cloned into an adenovirus shuttle vector (Ad-GFP, HanBio, Shanghai, China) carrying green fluorescent protein, namely, Ad-BMP2, Ad-SMAD7, and Ad-miR-181c-5p, respectively. The vector was then adopted for generation of recombinant adenovirus in HEK293 cells, with Ad-GFP as the control. The polybrene (TR-1003-G, 4-8 *μ*g/mL; Sigma) was added to enhance transfection efficiency.

### 2.9. Cell Counting Kit-8 (CCK8) Assay

The proliferative capacity of BMSCs was assessed employing the CCK8 kit (K1018, Apexbio, Boston, MA, USA). Cells were seeded into 96-well culture plates (1 × 10^3^ cells/well), and each well was added with 100 *μ*L of medium replenishing 10% FBS. After incubation for the indicated time, 10 *μ*L of CCK8 solution was added to each well and incubated at 37°C for 4 h. After incubation, optical density (OD) values were quantified at 450 nm and cell growth curves were constructed with the OD values on day 1, day 2, and day 3. Five parallel wells were prepared for each experiment.

### 2.10. Transwell Migration Assay

A total of 1 × 10^5^ cells cultured in serum-free medium were added to the apical Transwell chamber (BD Falcon, Franklin Lakes, NJ, USA), and the basolateral chamber was added with DMEM replenishing 10% FBS (600 *μ*L) for incubation for 8 h at 37°C. The cells in the apical chamber were removed, and the remaining cells on the surface of the Transwell bottom were fixed utilizing 4% paraformaldehyde. After staining with 0.2% crystalline violet for 15 min, the positive cells were observed under an inverted light microscope (CarlZeiss, Germany) and photographed, followed by counting utilizing ImageJ software.

### 2.11. Dual-Luciferase Reporter Gene Assay

The target binding site of SMAD7 with miR-181c-5p was inserted into the dual-luciferase reporter gene vector pGL3 promoter vector (Promega, Madison, WI, USA), followed by preparation of mutation vectors of the SMAD7 binding site sequence with the help of a point mutation kit (Takara, Japan), namely, PmirGLO-SMAD7-WT and PmirGLO-SMAD7-MUT. The reporter plasmids were cotransfected with the miR-181c-5p mimic and its NC into 293T cells for 48 h. Afterwards, the luciferase activity was quantified utilizing the Dual-Luciferase® Reporter Assay System (E1910, Promega).

### 2.12. RT-qPCR

Trizol (no. 16096020, Thermo Fisher Scientific, New York, USA) was adopted for total RNA extraction. Complementary DNA (cDNA) was reversely transcribed utilizing an RT kit (RR047A, Takara, Japan) for the detection of mRNA. To detect miRNA levels, cDNA was obtained by RT with the help of the PrimeScript™ miRNA RT-qPCR kit (B532451, Sangon Biotech, Shanghai, China). SYBR® Green (DRR081, Takara, Japan) reagents were employed for loading, and samples were subjected to RT-qPCR in a real-time fluorescent quantitative PCR instrument (ABI 7500, ABI, Foster City, CA, USA). Glyceraldehyde-3-phosphate dehydrogenase (GAPDH) was used as a normalizer for mRNA while U6 for miRNA. The primers used for amplification were prepared by General Biotechnology (Shanghai, China). Primer sequences are listed in Supplementary Table 1. The relative transcript levels of the target genes were calculated by means of the 2^−ΔΔCT^ method.

### 2.13. Western Blot

Cells and EVs were lysed in radio immunoprecipitation analysis lysis buffer (P0013B, Beyotime). Protein concentration determination was processed utilizing a bicinchoninic acid assay kit (A53226, Thermo Fisher Scientific, Rockford, IL, USA). Proteins were separated by sodium dodecyl sulphate polyacrylamide gel electrophoresis and transferred to polyvinylidene difluoride membranes (IPVH85R, Millipore, Germany). The membranes were blocked at ambient temperature for 1 h in 5% bovine serum albumin and were incubated with primary antibodies of BMP2 (ab14933, 1 : 1000, Abcam, UK), SMAD7 (sc-365846, 1 : 1000, Santa Cruz, CA, USA), collagen II (ab34712, 1 : 5000, Abcam), aggrecan (ab3778, 1 : 1000, Abcam), Sox9 (ab185966, 1/1000, Abcam), CD9 (ab236630, 1 : 1000, Abcam), CD63 (ab134045, 1 : 1000, Abcam), CD81 (ab79559, 1 : 1000, Abcam), GAPDH (ab181602, 1 : 5000, Abcam), and *β*-actin (ab8226, 1 : 5000, Abcam) overnight at 4°C. The membranes were then incubated with a horseradish peroxide-labeled goat anti-mouse secondary antibody (ab6808; 1 : 2000; Abcam) or goat anti-rabbit secondary antibody (1 : 5000, ab6721, Abcam) for 1 h at ambient temperature. Enhanced chemiluminescence working solution (Millipore) was applied for development. The greyscale quantification of each group of bands in the Western blot images was processed utilizing ImageJ analysis software. *β*-Actin acted as a normalizer for EV identification and GAPDH for the rest.

### 2.14. Ectopic Cartilage Formation in a Mouse Model

The animal protocol was ratified by animal ethics committee of the Second Hospital Affiliated to Zhejiang University School of Medicine. Athymic nude mice (nu/nu) (4-6 weeks old; females) were procured from Slac Biotechnology (Shanghai, China). Mice were treated with adenovirus-infected BMSCs aberrantly expressing BMP2, SAMD7, and/or miR-181c-5p, each for 24 h (*n* = 10). Besides, copolymer of polyethylene glycol citrate-co-N-isopropylacrylamide (PPCNg) was synthesized, and the PPCN powder was dissolved in PBS (100 mg/mL) and sterilized by filtering through a 0.22 *μ*m filter. BMSCs (5 × 10^6^) were resuspended with 100 *μ*L of PPCNg solution. Cell suspension was injected subcutaneously (5 × 10^6^/100 *μ*L) into the ventral side of the mice, three times per mouse.

After 4 weeks of subcutaneous injection in mice, mice were euthanized and ectopic tissue blocks were removed from the injection sites, followed by image through microcomputed tomography with X-ray imaging at 45 KV and 500 mA. The tissues were then prepared into paraffin-embedded sections for subsequent experimentations.

### 2.15. Hematoxylin and Eosin (H&E) Staining

Paraffin-embedded sections were dewaxed, hydrated, fixed at ambient temperature for 30 s, and then stained with hematoxylin for 60 s, followed by differentiation with 1% alcohol hydrochloride for 3 s. Sections were stained with eosin for 3 min and sealed with gum, followed by observation of the morphological structure of the cartilage tissue of the knee under a microscope (BX63, Olympus, Japan).

### 2.16. Alcian Blue Staining

After dewaxing and hydrating, the paraffin-embedded sections were acidified with 0.1 N hydrochloric acid for 5 min. The sections were then immersed in Alcian blue staining solution (B8438, Sigma) for 10 min, followed by 2 washes utilizing 0.1 N hydrochloric acid solution for 1 min each. Then, the sections were sealed with gum and air-dried. The morphological structure of the cartilage tissue of the knee joint was observed under a light microscope.

### 2.17. Immunohistochemical Staining (IHC)

Mouse cartilage tissue sections were baked at 60°C for 20 min, soaked in xylene for 15 min, dehydrated with ethanol of 100%, 95%, 90%, 85%, and 80%, and washed with double-distilled water for several times. The sections were immersed in 3% H_2_O_2_ at ambient temperature for 10 min to block endogenous peroxidase and subjected to antigen retrieval. After sealing with normal goat serum blocking solution (Sangon Biotech) for 20 min, the sections were probed overnight with the primary antibodies collagen II (ab34712, 1 : 50, Abcam), aggrecan (13880-1-AP, 1 : 200, Proteintech, Wuhan, China), and Sox9 (ab185966, 1 : 1000, Abcam) in the dark. Next, the sections were reprobed with the secondary antibody goat anti-rabbit IgG (ab6721, 1 : 1000, Abcam) for 30 min, followed by incubation with SABC (Vector Solutions, Inc., USA) at 37°C for 30 min. The sections were then developed with DAB (P0203, Beyotime) for 6 min. The sections were stained with hematoxylin for 30 s, dehydrated with gradient ethanol of 70%, 80%, 90%, and 95% and absolute ethanol for 2 min, respectively, cleared with xylene, mounted with neutral resin, and observed under the microscope (BX63, Olympus).

### 2.18. Statistical Analysis

SPSS 21.0 software (IBM, Armonk, NY, USA) was processed for data analysis. Measurement data were described in the form of mean ± standard deviation derived from at least 3 independent studies. The unpaired *t*-test was applied for the comparison between two groups, and one-way analysis of variance (ANOVA) with Tukey's post hoc test for comparison among multiple groups. Two-way ANOVA was carried out for data comparison at different time points. *p* < 0.05 indicated statistical significance.

## 3. Results

### 3.1. hUCMSC-EVs Can Be Internalized by BMSCs

To analyze the effect of hUCMSC-EVs on the biological functions of BMSCs, we first isolated and cultured hUCMSCs, and microscopic observation revealed that hUCMSCs exhibited a spindle-shaped and fibroblast-like morphology (Supplementary Figure 1A). Meanwhile, hUCMSCs had highly expressed positive markers CD151, CD73, and CD44 and lowly expressed negative markers CD34 and CD133 (Supplementary Figure 1B). Further, we found that the isolated hUCMSCs were able to differentiate into osteoblasts, chondrocytes, and adipocytes (Supplementary Figures 1C–1G). RT-qPCR revealed elevated expression of osteogenic-related factors RUNX2 and osterix, chondrogenic-related factors Sox9 and COL2, and adipogenetic-related factor PPAR-*γ* under different CM (Supplementary Figure 1H). These findings supported that hUCMSCs were successfully extracted.

**Figure 1:**
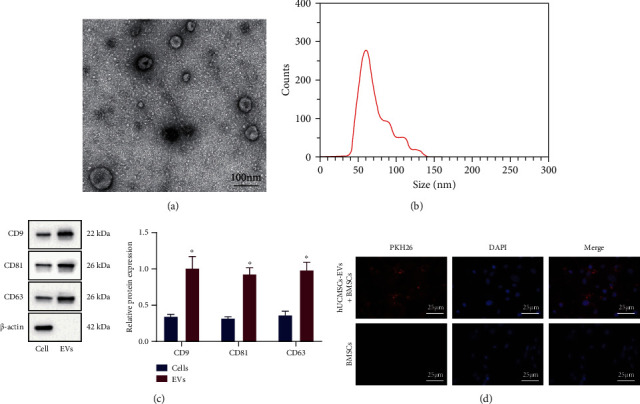
Isolation and identification of hUCMSC-EVs and observation of uptake of EVs by BMSCs. (a) EV morphology observed under the TEM (scale bar = 100 *μ*m). (b) EV particle size distribution detected by NTA. (c) The expression of CD9, CD81, CD63, and *β*-actin in hUCMSCs and hUCMSC-EVs measured by Western blot analysis. (d) After PKH26 labeling of hUCMSC-EVs, uptake of EVs by BMSCs was observed under fluorescence microscopy (scale bar = 25 *μ*m). ^∗^*p* < 0.05, compared with hUCMSCs.

Subsequently, we isolated the EVs from hUCMSCs using ultracentrifugation. Under TEM, hUCMSC-EVs exhibited a round or oval membranous vesicle-like morphology (Figure 1(a)). The diameter of EVs was about 50-150 nm (Figure 1(b)). Moreover, in hUCMSC-EVs, the levels of EVs surface markers CD9, CD81, and CD63 were significantly increased, while *β*-Actin was not expressed (Figure 1(c)). The above results confirmed the successful isolation of hUCMSC-EVs.

Next, BMSCs were incubated with PKH26- (red) labeled hUCMSC-EVs, and the uptake of EVs by hUCMSCs was observed under the microscope after 24 h. Red fluorescence was observed in BMSCs (Figure 1(d)). The above results indicated that BMSCs could uptake hUCMSC-EVs.

### 3.2. hUCMSC-EVs Promote the Proliferation, Migration, and Cartilage Differentiation Abilities of BMSCs

**Figure 2:**
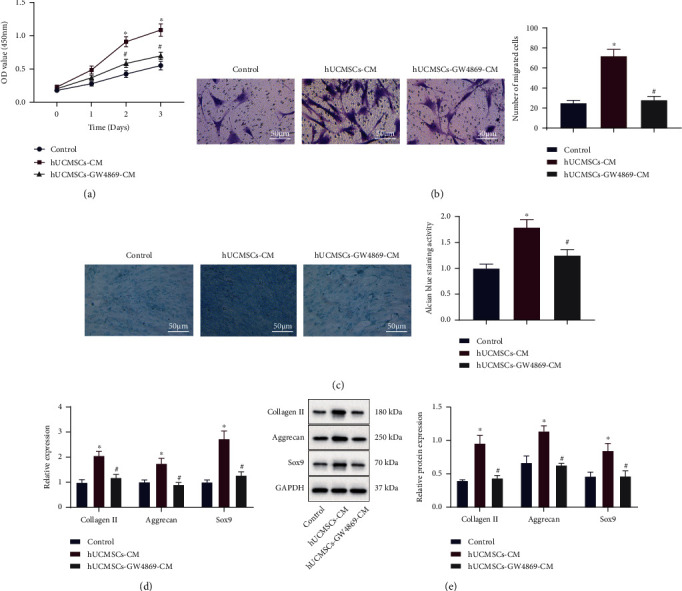
Effect of hUCMSC-EVs on the proliferation, migration, and cartilage differentiation of BMSCs. BMSCs were treated with hUCMSC-CM alone or combined with hUCMSC-GW4869-CM. (a) Proliferation of BMSCs was detected by the CCK8 assay. (b) Migration of BMSCs was detected by the Transwell assay (scale bar = 50 *μ*m). (c) Cartilage differentiation ability of BMSCs was detected by Alcian blue staining (scale bar = 50 *μ*m). (d) mRNA levels of collagen II, aggrecan, and Sox9 in BMSCs were determined by RT-qPCR. (e) Protein levels of collagen II, aggrecan, and Sox9 in BMSCs were measured by Western blot analysis. ^∗^*p* < 0.05, compared with the control; ^#^*p* < 0.05, compared with BMSCs treated with hUCMSC-CM. All experiments were repeated for three times.

To analyze the effect of EVs from hUCMSCs on the repair of cartilage injury, we treated hUCMSCs with GW4869 for 24 h and incubated BMSCs with the supernatant from CM. After coincubation with CM, the proliferative and migratory capacities of BMSCs were promoted, while those were suppressed after further addition of GW4869 (hUCMSC-GW4869-CM) (Figures 2(a) and 2(b)).

The chondrogenic ability of BMSCs was further enhanced after incubation with CM but curtailed after hUCMSC-GW4869-CM incubation (Figure 2(c)). As described by RT-qPCR and Western blot analysis, the expression of cartilage-specific genes collagen II, aggrecan, and Sox9 was significantly elevated in BMSCs coincubated with CM, while opposing tendency was detected after hUCMSC-GW4869-CM treatment (Figures 2(d) and 2(e)). Collectively, hUCMSC-EVs could be internalized by BMSCs to promote the proliferation, migration, and cartilage differentiation capacities of BMSCs.

### 3.3. hUCMSC-EVs Deliver miR-181c-5p to BMSCs to Promote BMSC Proliferation, Migration, and Cartilage Differentiation

**Figure 3:**
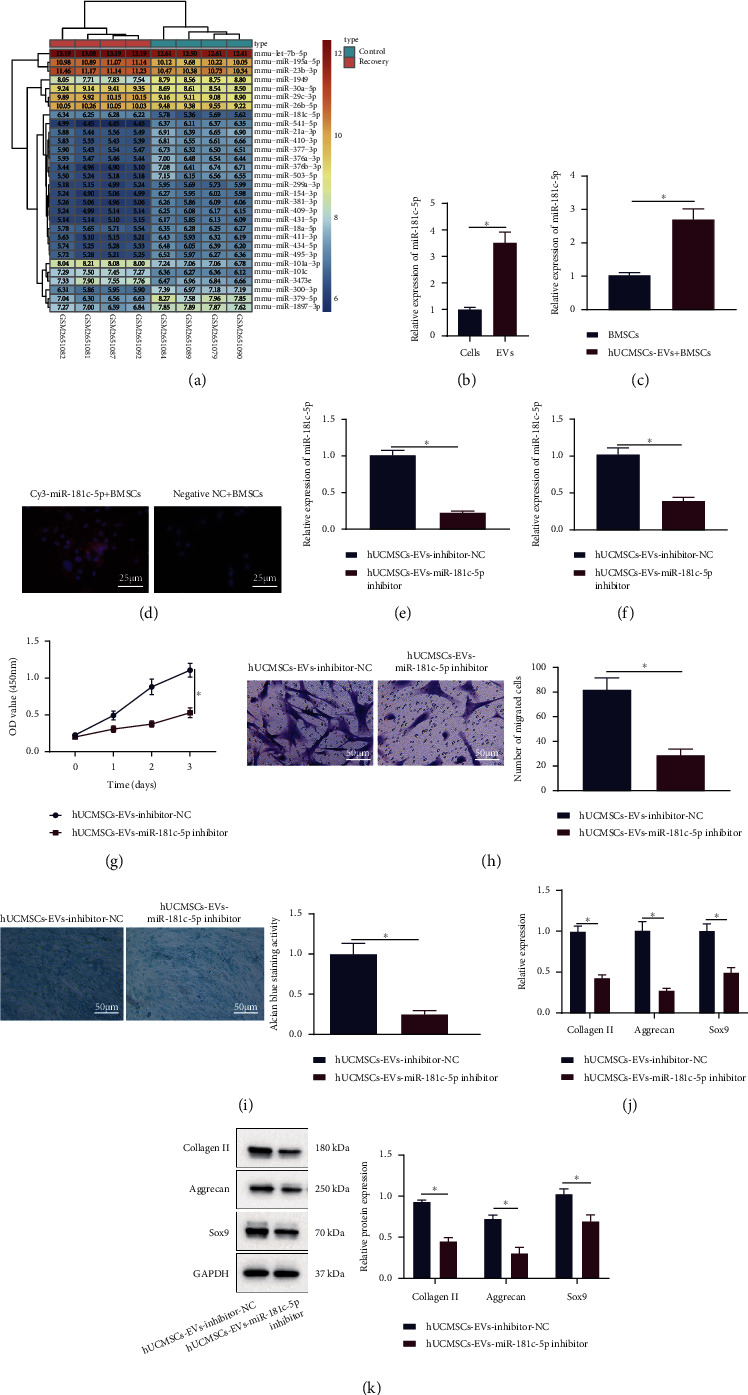
Effect of hUCMSC-EVs carrying miR-181c-5p on the proliferation, migration, and cartilage differentiation of BMSCs. (a) Heat map of expression of the top 30 miRNAs with the smallest *p* values in GSE99736. Color scale (blue to orange) indicates expression values from small to large (*n* = 3). (b) miR-181c-5p expression in hUCMSCs and their secreted EVs was determined by RT-qPCR. (c) miR-181c-5p expression in BMSCs after coincubation of hUCMSC-EVs was determined by RT-qPCR. (d) The fluorescence after treatment of Cy3-labeled hUCMSC-EV-miR-181c-5p (scale bar = 25 *μ*m). (e) RT-qPCR for miR-181c-5p expression in hUCMSC-EVs. BMSCs were coincubated with hUCMSC-EVs transduced with the miR-181c-5p inhibitor. (f) miR-181c-5p expression in BMSCs was determined by RT-qPCR. (g) Proliferation of BMSCs was detected by the CCK8 assay. (h) Migration of BMSCs was detected by the Transwell assay (scale bar = 50 *μ*m). (i) Cartilage differentiation ability of BMSCs was detected by Alcian blue staining (scale bar = 50 *μ*m). (j) mRNA levels of collagen II, aggrecan, and Sox9 in BMSCs were determined by RT-qPCR. (k) Protein levels of collagen II, aggrecan, and Sox9 in BMSCs were measured by Western blot analysis. ^∗^*p* < 0.05. All experiments were repeated for three times.

The molecular mechanisms by which hUCMSC-EVs promote the repair of cartilage injury were our next focus. Differential analysis on the microarray dataset GSE99736 yielded 93 differentially expressed miRNAs, and the heat map of expression of the top 30 miRNAs with the smallest *p* values was plotted (Figure 3(a)). miRNAs that were highly expressed in the recovery group were miR-101c, miR-101a-3p, miR-29c-3p, miR-195a-5p, miR-30a-5p, let-7b-5p, miR-23b-3p, miR-26b-5p, miR-181c-5p, and miR-3473e. Previous evidence has noted the elevated miR-181c-5p in hUCMSC-EVs [8]. Thus, we decided to further analyze the role of miR-181c-5p in the repair of cartilage injury.

As expected, miR-181c-5p was elevated in hUCMSC-EVs by RT-qPCR (Figure 3(b)). After coincubation of hUCMSC-EVs, elevated miR-181c-5p expression was also detected in the BMSCs (Figure 3(c)). BMSCs were coincubated with hUCMSCs expressing Cy3-miR-181c-5p, and we found that BMSCs showed red fluorescence, indicating that Cy3-labeled miR-181c-5p was internalized by BMSCs (Figure 3(d)). Thus, hUCMSC-EVs-miR-181c-5p could be internalized by BMSCs.

For analyzing the effect of hUCMSC-EV-miR-181c-5p on the repair of cartilage injury, EVs were extracted after downregulation of miR-181c-5p in hUCMSCs. RT-qPCR exhibited a decline in miR-181c-5p expression in hUCMSC-EVs (Figure 3(e)). Additionally, miR-181c-5p was downregulated in BMSCs coincubated with EVs from hUCMSCs transduced with the miR-181c-5p inhibitor (Figure 3(f)). Furthermore, inhibition of miR-181c-5p in EVs suppressed the proliferation, migratory, and chondrogenic abilities of BMSCs (Figures 3(g)–3(i)). Loss-of-function of miR-181c-5p in EVs further reduced the expression of collagen II, aggrecan, and Sox9 in BMSCs (Figures 3(j) and 3(k)).

These findings unveiled that hUCMSC-EVs delivered miR-181c-5p to BMSCs, which in turn promoted the proliferative, migratory, and cartilage differentiation potentials of BMSCs.

### 3.4. miR-181c-5p Inhibits SMAD7 Expression in BMSCs

**Figure 4:**
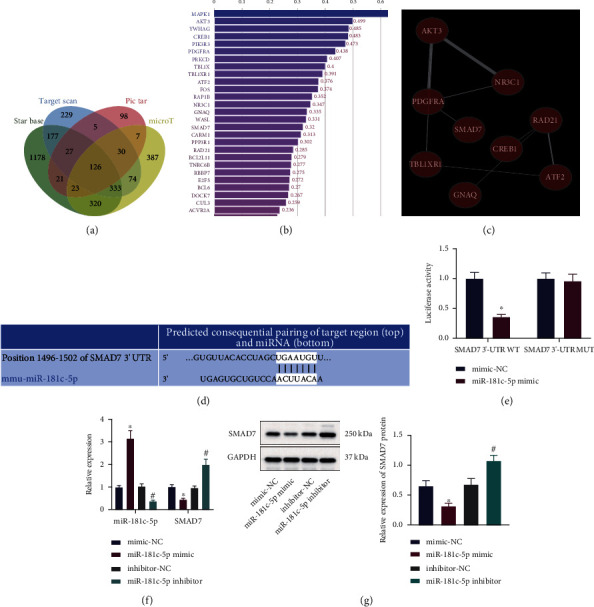
Regulation of SMAD7 expression by miR-181c-5p in BMSCs. (a) Venn diagram of the intersection of predictive results of the target genes of miR-181c-5p from StarBase, TargetScan, PicTar, and microT. (b) Correlation bar graph of candidate target genes and cartilage analyzed by using a PH&Enolyzer tool. (c) Network map of coexpression relationship of candidate genes analyzed by using a PH&Enolyzer tool. (d) The binding sites of miR-181c-5p and SMAD7 predicted by the TargetScan database. (e) The targeting relationship between miR-181c-5p and SMAD7 verified by the dual-luciferase reporter gene assay. (f) miR-181c-5p expression and SMAD7 mRNA level in BMSCs treated with the miR-181c-5p mimic or inhibitor determined by RT-qPCR. (g) SMAD7 protein level in BMSCs treated with the miR-181c-5p mimic or inhibitor measured by Western blot analysis. ^∗^*p* < 0.05, compared with BMSCs transduced with mimic-NC; ^#^*p* < 0.05, compared with BMSCs transduced with inhibitor-NC. All experiments were repeated for three times.

To further understand the mechanism of miR-181c-5p in hUCMSC-EVs affecting BMSCs, we used the miRNA target gene prediction tools StarBase, TargetScan, PicTar, and microT to predict the target genes of miR-181c-5p, respectively, which yielded 2205, 1001, 337, and 1300 target genes of miR-181c-5p, respectively. Following intersection, 126 candidate target genes were collected (Figure 4(a)). The correlation between candidate target genes and cartilage was analyzed by using the PH&Enolyzer tool (Figure 4(b)), and 20 genes including MAPK1, AKT3, and SMAD7 were found to have high scores according to the ranking of correlation scores. Further analysis of the coexpression relationship revealed that there was coexpression relationship among 9 genes including SMAD7 (Figure 4(c)). As previously reported, downregulation of SMAD7 promotes BMP2-induced chondrogenesis [11]. Therefore, it can be speculated that miR-181c-5p may inhibit SMAD7 to affect the BMP2-induced chondrogenesis.

The binding sites of miR-181c-5p and SMAD7 were subsequently predicted by the TargetScan database (Figure 4(d)), which was further verified by the luciferase assay that the luciferase activity was significantly inhibited in the cells cotransfected with the miR-181c-5p mimic and SMAD7 3′-UTR WT, while no significant difference was observed in the cotransfection of the miR-181c-5p mimic and SMAD7 3′-UTR MUT (Figure 4(e)). Furthermore, SMAD7 expression in BMSCs was significantly reduced after overexpression of miR-181c-5p, whereas loss-of-function of miR-181c-5p brought about an opposite trend (Figures 4(f) and 4(g)).

The obtained data demonstrated that miR-181c-5p could target the 3′-UTR region of the SMAD7 gene in BMSCs and repress SMAD7 expression.

### 3.5. miR-181c-5p Inhibits BMP2-Induced SMAD7 Expression in BMSCs

**Figure 5:**
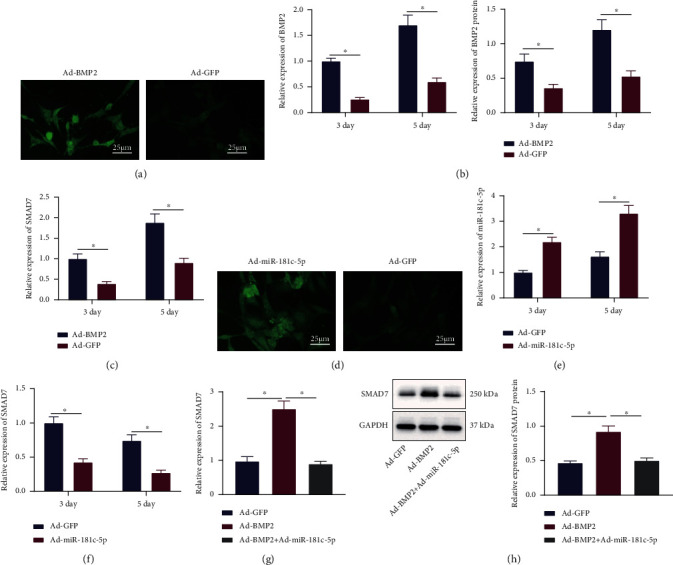
miR-181c-5p represses BMP2-induced SMAD7 expression. (a) Fluorescence microscopy observation of BMSCs after 3 days of infection with BMP2 overexpressing adenovirus (scale bar = 25 *μ*m). (b) BMP2 mRNA and protein levels in BMSCs after BMP2 overexpression determined by RT-qPCR and Western blot analysis. (c) SMAD7 mRNA level in BMSCs after BMP2 overexpression determined by RT-qPCR. (d) Fluorescence microscopy observation of BMSCs after 3 days of infection with miR-181c-5p adenovirus (scale bar = 25 *μ*m). (e) miR-181c-5p expression after overexpression of miR-181c-5p in BMSCs determined by RT-qPCR. (f) SMAD7 expression in BMSCs after overexpression of miR-181c-5p determined by RT-qPCR. (g) mRNA level of SMAD7 in BMSCs treated with Ad-BMP2 alone or combined with Ad-miR-181c-5p determined by RT-qPCR. (h) Protein level of SMAD7 in BMSCs treated with Ad-BMP2 alone or combined with Ad-miR-181c-5p measured by Western blot analysis. ^∗^*p* < 0.05. All experiments were repeated for three times.

Next, the regulatory mechanism of miR-181c-5p in BMP2-induced SMAD7 was investigated. Adenovirus infections of BMSCs overexpressing BMP2 (green fluorescence) were constructed, which showed that BMSCs had strong green fluorescence expression and significantly elevated expression of BMP2 and SMAD7 (Figures 5(a)–5(c)). After Ad-miR-181c-5p (green fluorescence) treatment, BMSCs had strong green fluorescence, and miR-181c-5p expression was distinctly increased while SMAD7 expression was decreased (Figure 5(d)–5(f)). Subsequently, we found that Ad-BMP2 treatment promoted SMAD7 expression, while further Ad-miR-181c-5p treatment suppressed SAMD7 expression (Figure 5(g) and 5(h)).

Conclusively, BMP2 promoted SMAD7 expression, while miR-181c-5p inhibited BMP2-induced SMAD7 expression.

### 3.6. miR-181c-5p Promotes BMP2-Induced Chondrogenesis by Suppressing SMAD7 Expression in BMSCs

**Figure 6:**
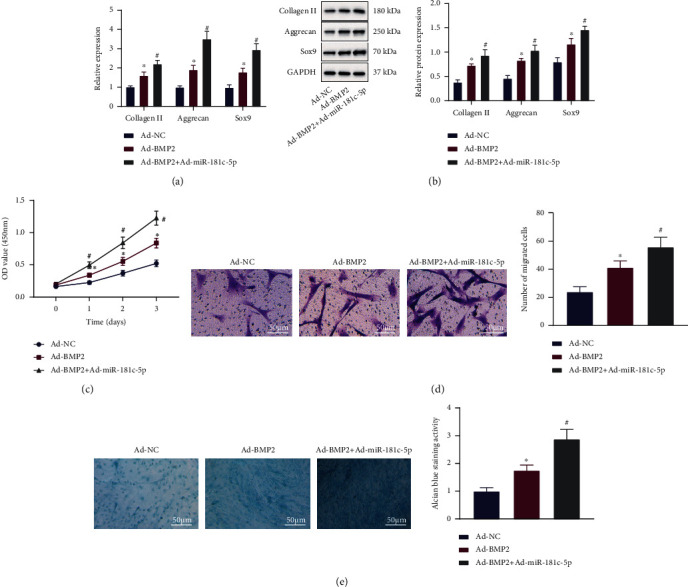
Effect of the BMP2/miR-181c-5p/SMAD7 axis on the proliferation, migration, and cartilage differentiation of BMSCs. BMSCs were treated with Ad-BMP2 alone or combined with Ad-miR-181c-5p. (a) mRNA levels of collagen II, aggrecan, and Sox9 in BMSCs determined by RT-qPCR. (b) Protein levels of collagen II, aggrecan, and Sox9 in BMSCs measured by Western blot analysis. (c) Proliferation of BMSCs detected by the CCK8 assay. (d) Migration of BMSCs detected by the Transwell assay (scale bar = 50 *μ*m). (e) Cartilage differentiation ability of BMSCs detected by Alcian blue staining (scale bar = 50 *μ*m). ^∗^*p* < 0.05, compared with BMSCs treated with Ad-NC; ^#^*p* < 0.05, compared with BMSCs treated with Ad-BMP2. All experiments were repeated for three times.

Next, we examined the effect of miR-181c-5p/SMAD7 expression on BMP2-induced cartilage injury repair using the cell model constructed above. Upon Ad-BMP2 treatment, the expression of collagen II, aggrecan, and Sox9 was significantly increased, which was further increased after both Ad-BMP2 and Ad-miR-181c-5p treatments (Figures 6(a) and 6(b)). BMSC proliferation, migratory, chondrogenic capabilities were enhanced after overexpression of BMP2, which were further enhanced after further overexpression of miR-181c-5p (Figures 6(c)–6(e)).

The aforementioned results demonstrated that miR-181c-5p could inhibit BMP2-induced SMAD7 expression and upregulate the expression of the cartilage-specific genes to promote the proliferative, migratory, and chondrogenic differentiation potentials of BMSCs.

### 3.7. Overexpression of BMP2 and miR-181c-5p Promotes Repair of Cartilage Injury

**Figure 7:**
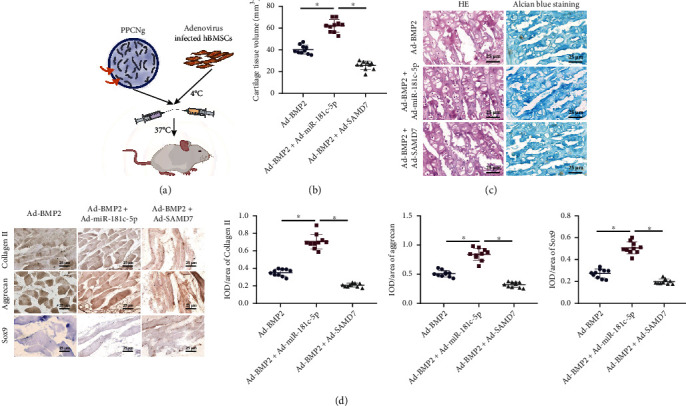
Effect of the BMP2/miR-181c-5p/SMAD7 axis on cartilage injury repair in mice. Mice were subcutaneously injected with PPCN-g hydrogel with BMSCs infected with Ad-SAMD7 or Ad-miR-181c-5p (*n* = 10). (a) Schematic diagram of the application of PPCN-g hydrogel and BMSCs to treat an animal model of ectopic osteogenesis. (b) Micro-CT scan to observe cartilage morphology. (c) Repair of cartilage injury detected by HE staining and Alcian blue staining (scale bar = 25 *μ*m). (d) Expression of collagen II, aggrecan, and Sox9 in cartilage tissues detected by immunohistochemistry (scale bar = 25 *μ*m). ^∗^*p* < 0.05.

The *in vivo* effect of miR-181c-5p on cartilage injury in hUCMSC-EVs was also investigated. We mixed PPCN-g hydrogel with BMSCs infected with Ad-SAMD7 or Ad-miR-181c-5p, which were then subcutaneously injected into mice (Figure 7(a)). Micro-CT scanning observation revealed that upon both Ad-BMP2 and Ad-SAMD7 treatments, a smaller volume of cartilage tissue was observed. However, after both Ad-BMP2 and Ad-miR-181c-5p treatments, the tissue volume increased (Figure 7(b)).

Furthermore, BMP2 induced chondrogenesis, which was significantly inhibited by overexpression of SMAD7. Overexpression of miR-181c-5p effectively promoted the formation of cartilage-like tissues and increased the number of chondrocytes, accompanied with a few hypertrophic chondrocytes (Figure 7(c)). Immunohistochemistry also revealed that expression of collagen II, aggrecan, and Sox9 was reduced after Ad-BMP2 and Ad-SAMD7 treatment, while opposing tendency was observed after Ad-BMP2 and Ad-miR-181c-5p treatment (Figure 7(d)).

Therefore, it could be concluded that BMP2 was able to induce the repair of cartilage injury, whereas overexpression of SMAD7 inhibited the repairing effect of BMP2, and overexpression of BMP2 and miR-181c-5p further promoted the BMP2-induced repair of cartilage injury.

## 4. Discussion

hUCMSCs are multipotent cells possessing self-renewal properties which are able to differentiate into osteocytes, adipocytes, and cartilage [14]. EVs are membranous vesicles released by almost all cell types, and hUCMSC-EVs bear great responsibility in tissue repair and regeneration [15, 16]. However, the underlying mechanism concerning the effect of hUCMSC-EVs in the repair of cartilage injury is rarely known, which deserves further analysis and study. Herein, we found through *in vitro* and *in vivo* assays that hUCMSC-EVs carrying miR-181c-5p could promote BMP2-induced repair of cartilage injury through suppression of SMAD7.

After isolation of EVs from hUCMSCs, we first confirmed the uptake of hUCMSC-EVs by BMSCs. In our flow cytometry analysis, CD151, CD73, and CD44 were highly expressed while CD34 and CD133 were poorly expressed. BMSCs are also positive for the cell surface markers CD13, CD44, CD73, CD90, CD105, and CD151 and negative for CD34 [17]. Moreover, EVs from BMSCs treated with glycoprotein nonmelanoma clone B can be taken up by BMSCs, which promoted the proliferation and osteogenic differentiation of BMSCs [18], which is partially consistent with our finding that hUCMSC-EVs could be internalized by BMSCs to promote the proliferative, migratory, and chondrogenic differentiation potentials of BMSCs. Furthermore, EVs from adipose-derived MSCs showed the most efficiency in the repair of cartilage injury and bone regeneration [19]. Another study has also indicated that chitosan oligosaccharides packaged into rat adipose MSC-derived EVs could promote cartilage injury repair and alleviate osteoarthritis [20]. MSC-derived EVs could alleviate osteoarthritis by regulating chondrocyte proliferation, migration, and apoptosis [21, 22]. Interestingly, chondrogenic EVs possess great potentials in accelerating chondrogenic differentiation and proliferation of hUCMSCs, which may be conducive to repair of articular cartilage [23]. These evidences supported the beneficial effects of hUCMSC-EVs on repair of cartilage injury, which led us to perform the study on the functional mechanism.

Moreover, the obtained data suggested that hUCMSC-EVs delivered miR-181c-5p to BMSCs to promote the cartilage differentiation of BMSCs. EVs can change the function properties of specific cells by delivering bioactive proteins and RNAs from their original cells [24]. As recently reported, EVs are secreted to mediate cell-to-cell communication and deliver genetic information by delivering miRNAs [25]. Meanwhile, miRNA delivery by EVs can be clinically applied for osteoarthritis treatment accompanied with loss of cartilage tissue homeostasis [26]. Ectopic expression of miR-181c is capable of limiting the proliferation of synoviocyte and expression of inflammatory factors correlated with the pathogenesis of osteoarthritis [9]. Furthermore, miR-181c accelerates BMSC proliferation and survival under oxidative stress injury [27]. However, how miR-181c-5p functioned in hUCMSC-EVs was little known based on the previous analysis. Thus, we further analyzed the downstream mechanism of miR-181c-5p, and SMAD7 was confirmed as its target gene. miR-181c-5p is implicated in pancreatic lineage commitment through direct inhibition of SMAD7 [28]. Similarly, SMAD7 functions as an important target gene for miR-181c in osteosarcoma cells [29]. Smad7 is a negative regulator of TGF-*β* signaling, which can be negatively regulated by miR-181c [30]. Additionally, SMAD7 is also an intracellular inhibitor of BMP signaling, and Smad7 can affect chondrogenesis by suppressing the BMP signaling [31]. BMP2 is a chondrogenic growth factor which is promising for cartilage tissue engineering [11]. Moreover, the BMP family can modulate the proliferation and differentiation of BMSCs to affect articular cartilage repair [32]. These findings substantiated our results that BMP2 was able to repair cartilage formation, whereas overexpression of SMAD7 inhibited the repairing effect of BMP2. Overexpression of BMP2 and miR-181c-5p further promoted BMP2-induced cartilage repair.

## 5. Conclusion

**Figure 8:**
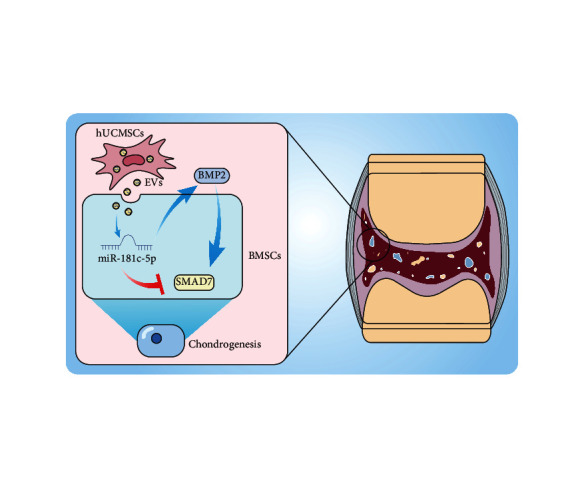
Molecular mechanism of hUCMSC-EVs carrying miR-181c-5p in affecting repair of cartilage injury through regulating BMP2-induced SMAD7 expression.

In conclusion, our study demonstrated that hUCMSC-EVs contributed to the induced proliferative, migratory, and chondrogenic differentiation potentials of BMSCs. This effect may be mediated by transferring miR-181c-5p to the BMSCs to inhibit its target gene SMAD7 expression, ultimately enhancing BMP2-induced repair of cartilage injury (Figure 8). Moreover, our findings offer novel insights into the intercellular communications between BMSCs and hUCMSC-EV-miRNAs.

## Data Availability

The datasets generated and/or analyzed during the current study are available from the corresponding author on reasonable request.

## Supplementary Materials

Supplementary Materials Supplementary Figure 1: isolation of hUCMSCs and identification of osteogenic, chondrogenic, and adipogenetic capacity. Supplementary Table 1 RT-qPCR primers.
