# Supplementary material for: Human Umbilical Cord Mesenchymal Stem Cell-Derived Extracellular Vesicles Carrying MicroRNA-181c-5p Promote BMP2-Induced Repair of Cartilage Injury through Inhibition of SMAD7 Expression
Source: Stem Cells Int. 2022 Jun 24;2022:1157498. doi: 10.1155/2022/1157498 (PMC9249498; doi:10.1155/2022/1157498)
Supplement: Supplementary Materials — Supplementary Figure 1: isolation of hUCMSCs and identification of osteogenic, chondrogenic, and adipogenetic capacity. Supplementary Table 1 RT-qPCR primers. [file 1157498.f1.docx]

**Supplementary Materials**

**
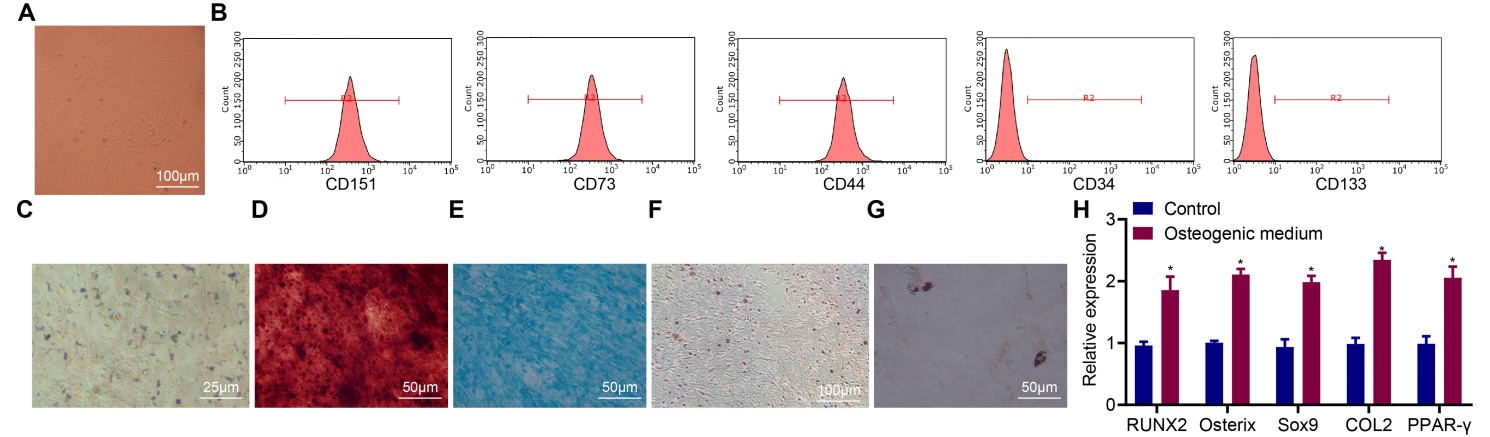
**

**SUPPLEMENTARY FIGURE 1** Isolation of hUCMSCs and identification of osteogenic, chondrogenic and adipogenesis capacity. A, Microscopic morphology of primary hUCMSCs after adherent under light microscope (scale bar = 100 μm). B, Flow cytometry for the surface markers of hUCMSCs. C, ALP detection of osteogenic differentiation ability of hUCMSCs (scale bar = 25 μm). D, Alizarin red staining detection the osteogenic differentiation ability of hUCMSCs (scale bar = 50 μm). E, Alcian blue staining for glycosaminoglycan synthesis to confirm hUCMSCs chondrogenic ability (scale bar = 50 μm). F, Immunohistochemistry for COL2 expression determination to confirm chondrogenic ability of hUCMSCs (scale bar = 100 μm). G, Oil red O staining to detect the adipogenesis ability of hUCMSCs (scale bar = 50 μm). H, RT-qPCR to detect the mRNA expression of osteogenic, chondrogenic and adipogenesis related factors. * *p* < 0.05.

**SUPPLEMENTARY TABLE 1** RT-qPCR primers

| Gene | Primer |
| --- | --- |
| miR-181c-5p (human) | F: 5ʹ-AACATTCAACCTGTCGGTGAGT-3’ |
|  | R: Reverse universal primer |
| U6 (human) | F: 5ʹ-CTCGCTTCGGCAGCACA-3’ |
|  | R: Reverse universal primer |
| SMAD7 (human) | F: 5’-TCTCCCCACCCCCAAATTAAG-3’ |
|  | R: 5′-TTCCTACATGGAAGGTCCGC-3’ |
| Collagen II (human) | F: 5′-CCAGATGACCTTCCTACGCC-3’ |
|  | R: 5′-TTCAGGGCAGTGTACGTGAAC-3’ |
| Sox9 (human) | F: 5′-GCTCTGGAGACTTCTGAACGA-3’ |
|  | R: 5′-CCGTTCTTCACCGACTTCCT-3’ |
| Aggrecan (human) | F: 5′-AGTCCTCAAGCCTCCTGTACTCA-3’ |
|  | R: 5′-GCAGTTGATTCTGATTCACGTTTC-3’ |
| GAPDH (human) | F: 5′-CTACACTGAGGACCAGGTTGTCT-3’ |
|  | R: 5′-TTGTCATACCAGGAAATGAGCTT-3’ |

Notes: RT-qPCR, reverse transcription quantitative polymerase chain reaction; miR-181c-5p, microRNA-181c-5p; SMAD7, small mother against decapentaplegic 7; GAPDH, glyceraldehyde-3-phosphate dehydrogenase
